# Patients’ perception about risks and benefits of antithrombotic treatment for the prevention of venous thromboembolism (VTE) after orthopedic surgery: a qualitative study

**DOI:** 10.1186/s12891-015-0777-x

**Published:** 2015-10-26

**Authors:** M. Najafzadeh, S. C. Kim, C. Patterson, S. Schneeweiss, J. N. Katz, G.W. Brick, J. E. Ready, J.M. Polinski, E. Patorno

**Affiliations:** Division of Pharmacoepidemiology and Pharmacoeconomics, Department of Medicine, Brigham and Women’s Hospital, Harvard Medical School, Boston, MA USA; Division of Rheumatology, Immunology and Allergy, Department of Medicine, Brigham and Women’s Hospital, Harvard Medical School, Boston, USA; Department of Orthopedic Surgery, Brigham and Women’s Hospital, Harvard Medical School, Boston, USA

**Keywords:** Antithrombotic agents, Benefit-risk assessment, Orthopedics, Patient understanding, Shared decision making, Venous thromboembolism

## Abstract

**Background:**

The 9th edition of the American College of Chest Physicians’ Antithrombotic Therapy and Prevention of Thrombosis guidelines emphasize the importance of considering the risk–benefit ratio of “patient-important” outcomes. However, little is known about patients’ perception and understanding regarding the different outcomes of antithrombotic treatment after orthopedic surgery, and the factors that influence their decision to use these treatments. Using a series of semi-structured interviews, we explored patients’ understanding and perception concerning the benefits and risks of antithrombotic treatment for the prevention of venous thromboembolism (VTE) after joint replacement surgery.

**Methods:**

A series of semi-structured interviews were conducted with patients who had undergone knee or hip replacement surgery at a tertiary care hospital (Brigham and Women’s Hospital, Boston, MA) in 2014. Discussions were recorded and transcribed. Two investigators independently coded and analyzed the data to identify important themes and concepts using the constant comparative method.

**Results:**

Of 64 patients who were invited, 12 patients (19 %) completed the interviews. The majority of patients (92 %) were aware of the benefits of antithrombotic therapy for reducing the risk of blood clots, while less than half of them had a clear understanding of deep vein thrombosis and pulmonary embolism. While all patients were aware of risk of minor bleeding, only 6 patients (50 %) considered the risk of major bleeding as a possible side effect of antithrombotic treatment. Overall, patients perceived bleeding as a less important outcome than a thrombotic event. The lack of awareness about the risk of major bleeding, the assumption that a short-term exposure would not meaningfully affect bleeding risk, and the assumption that bleeding is a controllable event influenced their perception. Most patients (83 %) stated that their decision to use antithrombotic medications was mainly based on the trust in their physician’s expertise.

**Conclusions:**

Patients perceived thrombotic events as more important outcomes than bleeding events. Patients’ understanding of thrombotic and bleeding events varies and may play a key role in their preferences. The majority of patients stated that trust in their physician’s expertise had a large influence on their decision to use antithrombotic medications.

## Background

Patients who undergo major orthopedic surgery (e.g. hip or knee replacement) are at risk of venous thromboembolism (VTE), which includes deep vein thrombosis (DVT) and pulmonary embolism (PE). The cumulative 35-day post-operative risks of symptomatic VTE, DVT, and PE are estimated to be as high as 4.3 %, 2.8 %, and 1.5 %, respectively [1]. Prophylactic antithrombotic treatment has been shown to decrease these risks to 1.8 %, 1.55 %, and 0.55 %, for VTE, DVT and PE respectively [1]. Although the results of meta-analyses of randomized clinical trials strongly support the benefit of prophylactic anticoagulation for reducing the risk of symptomatic VTE and fatal PE, the evidence concerning bleeding risk associated with prophylactic anticoagulation is not consistent [2, 3]. The recommendations of the 9th edition of the American College of Chest Physicians (ACCP) Guidelines on Antithrombotic Therapy and Prevention of Thrombosis emphasize the importance of considering the risk–benefit ratio of “patient-important outcomes”. These guidelines suggest that benefits of prophylactic antithrombotic treatment outweigh potential risks only in patients who are at sufficiently high risk of developing symptomatic VTE [4, 5].

Physicians’ decisions about using prophylactic treatment are usually based on the patient’s risk for developing VTE, expected benefits and risks of antithrombotic drugs, and factors such as treatment modality and duration. An implicit element in this process is the physicians’ awareness about outcomes and factors that are important for patients when deciding about antithrombotic therapy. However, there is little information about patients’ understanding and perception of the benefits, risks, and other factors that can influence their preferences for using or not using antithrombotic therapy [1]. Such information would be particularly valuable given the consistently reported underuse of antithrombotic medications [6, 7]. Exploring patients’ perceptions and understanding of different outcomes associated with antithrombotic therapy may provide useful information to physicians for making more patient-centered prescribing decisions. In this exploratory study, we investigated patients’ understanding and perception in regard to the benefits and risks of antithrombotic drugs for prevention of VTE after a joint replacement surgery using a qualitative approach.

## Methods

### Participants

This study involved a series of in-person or phone interviews with patients who had recently undergone knee or hip replacement surgery at a tertiary care hospital (Brigham and Women’s Hospital, Boston, MA). Using the hospital’s centralized clinical data registry (the Partners’ Research Patient Data Registry (RPDR)), we identified an initial list of patients who had undergone hip or knee replacement surgery between January and June 2014, and who were 18 years of age or older. Through the registry, we gathered data on the characteristics and the contact information of patients who had undergone surgery with one of two orthopedic surgeons that were part of the research team. Upon receiving their surgeons’ permission, patients were invited by mail to participate in a 30-min interview. The invitation letter briefly described the purpose of the study. The letter also stated that patients could refuse to answer any question or decide to withdraw at any point during the study period without any impact on the care that they receive from the hospital. All participants were offered a $50 honorarium in the form of a gift card upon completion of the interview. Patients who responded positively to our invitation were contacted by our study coordinator to arrange an interview time. Interviewing was planned to be discontinued once 15 patients agreed to participate or whenever the study team concluded that no new themes or concepts would arise during the interviews.

To ensure adequate time for reviewing, study information and fact sheets were sent to participants at least one week prior to conducting the interview and participants provided their consent at the beginning of each session. The protocol for this study was approved by the Behavioral Research Ethics Board of the Brigham and Women’s Hospital.

### Interview procedure

Patients initially were invited for in-person interviews at the tertiary care hospital. In a follow-up contact they were also offered the option to interview via phone. We used a semi-structured interview approach with a list of questions to guide discussion during the interviews (Please see Appendix). These questions were designed to address our study objective. In particular, we asked whether patients were aware and had clear understanding of potential complications that might occur following the surgical procedure, e.g. DVT and PE, in contrast with unrelated but possibly more familiar conditions, e.g. stroke and myocardial infarction (MI); whether they knew about the benefits and risks of using “blood thinners”; and what factors affected their decisions to comply with (or not comply with) prophylactic antithrombotic treatment. In addition to these predefined questions, both patients and interviewer had the opportunity to discuss issues that they deemed to be relevant to the study topic as they emerged during the interviews.

### Analysis

All discussions were digitally recorded, transcribed, and analyzed using the constant comparative method [8–10]. Transcripts were initially reviewed with the aim of developing an overall understanding of the scope and content of data. We identified issues requiring further clarification, which were included as discussion topics in the subsequent interviews. Subsequently, a line-by-line analysis of transcripts was conducted and codes were assigned to phrases and sentences as a concept became apparent. The appropriateness of the code assignments was assessed by reviewing the previously coded data and ascertaining consistent assignment of codes to concepts. As more data were reviewed, the code structure was modified inductively by refining the existing codes and adding new codes when necessary. Data were hand-coded and reviewed separately by two investigators (MN and CP) to identify major concepts and emerging themes. All discrepancies were resolved by in depth discussion of issues among three investigators (MN, CP, and EP).

## Results

Of 64 patients who were invited for interviews, 12 patients (19 %) responded back and all completed an interview (five in-person and seven phone interviews). Table 1 provides the demographic characteristics of the patients who were invited and those who participated in interviews. Patients who were 65 years and older and females were more likely to participate in the interviews. Race and primary insurance seem to be well balanced across patients who participated and did not participate in interviews.

**Table 1:** Characteristics of patients who were contacted and participated in the interviews

	Patients who participated in interviews (*n* = 12)		Patients who were invited but did not participate in interviews (*n* = 52)	
	n	%	n	%
Age (years)				
≤65	4	33 %	26	50 %
65-69	3	25 %	9	17 %
70-75	2	17 %	12	23 %
75-80	1	8 %	3	6 %
≥80	2	17 %	2	4 %
Female	9	75 %	23	44 %
Race (Black or African American)	1	8 %	6	12 %
Medicare as primary insurance	7	58 %	25	48 %
Type of surgery				
Hip replacement	9	75 %		
Knee replacement	3	25 %		
Prescribed antithrombotic medicatixon				
Warfarin	9	75 %		
Heparin	1	8 %		
Unnamed oral antithrombotic medication	2	17 %		

### Patients’ understanding of VTE

Among the 12 patients who completed interviews, 8 (67 %) stated that they were informed about potential complications (e.g. risk of blood clots) prior to surgery (Table 2). Only 6 patients (50 %) and 5 patients (42 %) had a clear understanding of DVT and PE, respectively, and described those conditions as being “a clot that forms in one of your veins that could break off and travel through the venous system or land in your lung” (Patient 103). In contrast, all of the patients in our sample had a basic understanding of stroke and MI, and were able to describe these conditions as the “loss of control of parts of the body or drooping of the face” (Patient 102), and “pain that feels like indigestion, you can have pain that starts in your chest and radiates out into I think it’s your left arm”. (Patient 112)

**Table 2:** Understanding of patients about different aspects of VTE prophylaxis who completed interviews (*n* = 12)

	n	%
Were informed about benefits and risks of prophylactic treatment prior to surgery	8	67 %
Were able to understand the explanations given by physicians (self reported)	11	92 %
Had sufficient understanding^a^ of PE	6	50 %
Had sufficient understanding of DVT	6	50 %
Had sufficient understanding of stroke and MI	12	100 %
Injection was a concern	3	25 %
Taking pills was a concern	1	8 %
Monitoring for INR was a concern	2	17 %
Considered major bleeding events as possible side effect of prophylactic treatment	6	50 %

VTE: venous thromboembolism; PE: Pulmonary embolism; DVT: deep vein thrombosis

^a^Sufficient understanding was defined as knowledge about key symptoms and general prognosis of PE, DVT, stroke, and MI

### Patients’ perceptions about the benefits of antithrombotic therapy

Eleven patients out of 12 (92 %) were aware of the benefits of antithrombotic therapy. These were generally described in terms of a reduction in the risk of blood clot formation after orthopedic surgery due to thinning of the blood. However, 7 out of 12 patients (58 %) assumed that prophylactic antithrombotic therapy could also reduce the risk of stroke and MI:

*“I know the benefits are it thins the blood. Therefore it makes it harder for a clot to form.” (Patient 102)*

*“Not having a stroke or a heart attack or a pulmonary embolism! Or having a clot so big that it blocks your leg. Then you have to have it taken care of.” (Patient 104)*

*“I know that the blood clot was a strong possibility after something like that and that you needed to take a blood thinner.” (Patient 106)*

### Patients’ perceptions about the risks of antithrombotic therapy

While all patients mentioned the risk of excess bleeding in case of injury and bruising as a possible side effect of antithrombotic treatments, only 6 patients (50 %) considered the risk of major bleeding events. Patients, in general, described the risk of bleeding associated with antithrombotic therapies as a consequence of “their blood becoming too thin”:

*“I know; I’m primarily familiar with the risks. I know that you are more prone to bruising and bleeding if you cut yourself at all or like injure yourself at all.” (Patient 109)*

*“The risk would be that you’d bleed a lot if you got cut. But other than that I don’t know.” (Patient 102)*

Only half of patients acknowledged serious bleeding as a possible side effect of antithrombotic therapy:

*“Well, [INR] has to be kept at a certain level or else your blood’s too thin. I guess you could have a cerebral hemorrhage or some other kind of bleeding.” (Patient 103)*

*“I do know that they can make you bleed too much. That’s one of the biggest. Sometimes you don’t know because it can be internal you know and you can bleed into your gut.” (Patient 104)*

*“I know that there’s a risk of bleeding internally and externally and not being able to stop it when you’re on Coumadin. And that things like cuts and falls that are very routine can be very serious when you’re on Coumadin.” (Patient 112)*

*“There are a lot of side effects. But my dad was on it and you know he would bleed terribly. He fell once and cut a small artery in his lip. Oh, my lord, he bled like crazy. I mean it thins out your blood. I did not have as much a problem with it myself. I didn’t bruise or anything. So I don’t know what the difference is.” (Patient 113)*

### Factors influencing patients’ decision to use antithrombotic medications

All patients (100 %) stated that they used antithrombotic medications as prescribed by their physicians. Most patients (83 %) stated that their decision to use the prescribed antithrombotic medications was mainly based on the trust in their physician’s expertise:

*“If I’m going to let him go in there and take a piece of my hip and replace it with a piece of plastic and metal and he recommends that I take blood thinners, I’m going to say yes.” (Patient 102)*

*“I took them because my doctor advised it. And I had a lot of confidence in him based on my primary care physician, her very high recommendation.” (Patient 112)*

Patients also reported that they felt that the risk of bleeding was not substantially high since antithrombotic therapy only lasted for a short time period:

*“I don’t feel that I was at particular risk taking them if that’s part of it…I was only on them for a relatively short time.” (Patient 104)*

*“It was broached to me as a problem that would last for a several-week period, not a long-term problem.” (Patient 102)*

*Was I [worried about risks]? No. Because I knew I wouldn’t be on them that long. If I was on them a long time, yes. Some people are on blood thinners for a long time.” (Patient 104)*

Patients reported that they perceived bleeding as an adverse event that could be monitored, and therefore could be controlled and reversed by stopping or modifying the antithrombotic medication being taken. Consequently, these patients believed that bleeding events result in less severe consequences compared to blood clots:

*“They measured my blood every day or every other day… if it was the least bit up or down, they said take an extra pill, or don’t take any for two days and then we’ll do it again. You know it was monitored. So I didn’t feel as if I was ever at risk because I was being monitored. If I was [at] high enough [risk of] major bleeding they would have found it immediately and taken me off the medicine; So I felt safe that I was being followed.” (Patient 102)*

*“I feel like the high bleeding is more manageable or more easily treatable than the risks of a blood clot.” (Patient 109)*

Overall, patients appeared to consider the risk of blood clots and their consequences to be more significant than the risk of bleeding events. Eight patients (67 %) explicitly stated that they were willing to trade-off an increased risk of bleeding for a reduced risk of thrombotic events:

*“I think I would go with the higher bleeding risk. It just seems to make sense … A high bleeding risk…I can stop taking Celebrex if I have to. I could do that and lower my risk of bleeding.” (Patient 102)*

*“The blood clot is so serious. I wouldn’t want to take that chance. Although my mother did die of a cerebral hemorrhage so I did have some concern on the other side about bleeding. But I think that’s a lesser risk than the blood clot if you have to weigh the two.” (Patient 103)*

*“So I think [bleeding] would’ve been the lesser of the evils so to speak.” (Patient 106)*

*“For me probably more the concern was the bleeding risk…maybe that was worrying me more than the blood clot risk…But I think I trust my doctor… [and] what they think is the right thing for me. I was more concerned with the bleeding …I thought maybe if it’s not long-term, it’s going to benefit me rather than harm me.” (Patient 107)*

A few patients stated that they were more concerned about bleeding risk compared to risk of blood clots and embolism. However, even the patients that did have legitimate concerns (e.g. family bleeding history, blood disorders) reported that they did not discuss these concerns with their doctors. Therefore, these patients followed their physician’s advice to use antithrombotic medications under the assumption that no other option was available and that their physician had carefully considered the patient’s individual profile to assure that the benefits outweighed the risks:

*“I mean I would think that a doctor would be making a decision like that based on the individual patient.” (Patient 109)*

*“I just took it because I thought they know better so it means I can take it. But I was a little concerned with the ITP [Idiopathic thrombocytopenic purpura] … I didn’t really question the ulcer, that I had an ulcer before in the stomach…the ITP was the main [concern]. ..You say “Okay, you know where my health issues are.” I think at that point I trust what he decides for me.” Patient 107*

Overall, treatment modality (e.g. injection vs. pills), need for frequent monitoring, diet requirements to control potential interactions with antithrombotic medications, and cost played minimal role in patients’ willingness to use antithrombotic medications after surgery.

## Discussion

Physicians’ awareness of patients’ understanding and preferences regarding the benefits and risks of antithrombotic treatment can enrich the patient-physician communication process and increase the likelihood of making optimal treatment decisions. The large variety of therapeutic options available for thromboprophylaxis following orthopedic surgery, which include low molecular-weight heparin, fondaparinux, low-dose unfractionated heparin, warfarin, new oral anticoagulants, aspirin, and mechanical compression devices [ACCP 2012] [1], and the lack of clarity regarding which prophylactic strategy (or strategies) is/are optimal or suboptimal [AAOS, 2012] [11], suggests that the knowledge about patients’ understanding and preferences could play a particularly relevant role in the prescribing decision. Using a qualitative approach, we investigated the understanding that patients have regarding VTEs such as PE and DVT, and the factors that can influence patients’ decisions for using (or not using) thromboprophylactic medications that they have been prescribed.

Our results suggest that patients in our sample were aware of the increased risk of thrombotic events after orthopedic surgery and were able to describe the benefits of antithrombotic treatment in terms of a reduction in the risk of thrombosis by “thinning” their blood. However, less than half of patients in our sample had a clear understanding of DVT and PE. The majority of patients in our sample assumed that antithrombotic treatment can also reduce risk of stroke and MI, a benefit that has not been substantiated by evidence. Although patients were generally familiar with the concept of bleeding as a side effect of treatment, only half of them acknowledged the possible occurrence of serious bleeding. Treatment modality, need for monitoring, drug-food interaction, and cost did not seem to have a large influence on patients’ use of thromboprophylaxis in our sample.

Overall, patients perceived bleeding as a less important occurrence compared to a thrombotic event and were willing to trade additional bleeding risk in exchange for a reduction in the risk of thrombotic events by complying with the prescribed prophylactic strategy. They generally viewed bleeding as a minor event that could be monitored and controlled if necessary. This is despite the fact that mortality rates as a result of PE and major bleeding in patients undergoing hip and knee replacement surgery are largely unknown [4]. Patients also believed that the risk of bleeding was not significant because prophylactic antithrombotic treatment was only prescribed for a short period of time. This suggests a differential perception for the impact of treatment duration on bleeding versus a thromboembolic event. Patients generally believed that their physician had carefully considered all the risks and benefits and the individual patient’s profile in their prescription decisions. Therefore, the large majority of patients followed their physician’s recommendation, regardless of their individual perception about risks, benefits, and other aspects of treatment. This finding is line with several previous studies that have shown patient trust in their doctors to have strong influence on medication use and adherence [12–14].

Surgeons’ attitudes towards VTE prophylaxis in surgical patients have been explored in a study by Polk et al. [15]. Their results suggested that surgeons’ opinions varied widely based on practice sites, modes, and specialty. However, the same study found that orthopedic and gynecologic surgeons had less diverse opinions and tended to agree with each other in terms of the benefit of post-surgical thromboprophylaxis, while greater variation occurred among general and trauma surgeons. The literature about patient preferences concerning VTE prophylaxis is sparse [16, 17]. The 9th edition of the American College of Chest Physicians (ACCP) Guidelines on Antithrombotic Therapy and Prevention of Thrombosis emphasize the importance of considering “patient-important outcomes,” and has used the results of a systematic review on patient values and preferences for antithrombotic therapy to inform these guidelines [18, 19]. However, these studies reflect patients’ values and preferences around the prevention of thrombosis, stroke and MI, but do not specifically explore VTE, DVT, or PE. Only one study by Wong et al. [20] has recently explored patients’ preferences regarding VTE prophylaxis within the United States. In this study, a sample of 227 patients hospitalized in surgical and medical units in an academic medical center, and with a recent prescription for thromboprophylactic medications, completed a survey to assess whether they preferred injectable versus oral medications. Based on the findings that suggested a preference for oral medications, the authors concluded that tailoring treatment route to patient preferences might improve compliance with thromboprophylactic therapy. This study focused solely on patient preferences with respect to treatment modality, and did not explore the relative importance of other factors on patients’ decisions to use these treatments. In our study, we found that patients’ trust in their physician’s recommendation and personal considerations regarding the overall balance between the risks and benefits of VTE prophylaxis are the main determinants of compliance. Conversely, treatment modality had little influence on patients’ decisions in our study.

Our study has several limitations. First, our sample was selected from a major academic hospital in an urban setting and all patients interviewed underwent surgery with either one of two surgeons. Therefore, our results may not be generalizable to other types of practices and settings. Second, our small sample size may limit the generalizability of our findings. However, after interviewing 12 patients, we observed no new theme or concept emerging during the interviews. Third, all interviews were conducted post-surgery and therefore, patients’ preferences may have been influenced by their experience. Third, our findings could be influenced by recall bias as some patients may forget the details of their interaction and communication with their care providers over time. However, in order to reduce chances of recall bias, we only invited patients who had undergone a surgery within six month prior to interview.

This study extends the current knowledge about patients’ understanding of VTEs and their consequences, as well as patients’ perceptions regarding the benefits, risks, and inconveniences associated with thromboprophylactic therapy. The results of this study provide preliminary important information to inform future research aimed at quantitatively measuring patient preferences and the relative impact of different factors on patients’ willingness to use VTE thromboprophylaxis, which will ultimately assist physicians in making more patient-centered prescribing decisions.

## Conclusions

Patients perceived thrombotic events as more important outcomes than bleeding events. Patients’ understanding of thrombotic and bleeding events varies and may play a key role in their preferences. The majority of patients stated that trust in their physician’s expertise had a large influence on their decision to use antithrombotic medications.

## Data availability

To access anonymized data please contact. Najafzadeh (mnajafzadeh@bwh.harvard.edu).

## Appendix

### Interview Format

1. **Introduction and purpose of the study**
2. **Our study team**
  - Interviewer will provide a brief explanation of the purpose of our study.
3. **Open-ended questions**
  - The interviewer will introduce the study team, affiliations, and sources of funding.

Given that you have recently had a knee or hip replacement surgery, are you aware of any complications that may occur after the surgery?

Has your physician explained to you what are the possible problems that may happen after hip or knee replacement surgery? Were you able to clearly understand those explanations?

Have you ever heard the terms “pulmonary embolism” or “deep vein thrombosis”? How do you compare those problems with stroke or heart attack?

Has your physician discussed with you options to prevent these complications? Has he prescribed any blood thinners (for example, heparin, warfarin, dabigatran, aspirin)? Has he discussed the use of any pressure devices (for example, inflatable air sleeves, or elastic compression stockings)?

Do you know what kind of benefits or risks blood thinning medications can have? Have you informed your doctor of any questions or concerns you had before starting these medications?

If you have been prescribed any of these medications, have you used them as your physician suggested? Have you informed your doctor of any questions or concerns after starting these medications?

What are the factors that can influence your decision whether to use (or not to use) these medications?

Do you consider any of the following issues as something that can influence your decision to take these medications?

I am not sure about the benefits of these medications

I am not sure about the side effects of these medications (bleeding, PE, DVT, stroke, MI)

I dislike injections

I dislike taking several pills per day

The cost is an issue for me

## Abbreviations

VTE: Venous thromboembolism
DVT: Deep vein thrombosis
PE: Pulmonary embolism
MI: Myocardial infarction
